# Organizational Justice and Refraining from Seeking Medical Care Among Japanese Employees: A 1-Year Prospective Cohort Study

**DOI:** 10.1007/s12529-018-9756-6

**Published:** 2018-11-27

**Authors:** Akiomi Inoue, Akizumi Tsutsumi, Hisashi Eguchi, Norito Kawakami

**Affiliations:** 1 Department of Public Health, Kitasato University School of Medicine, 1-15-1 Kitasato, Minami-ku, Sagamihara, 252-0374 Japan; 2 Department of Mental Health, Graduate School of Medicine, The University of Tokyo, 7-3-1 Hongo, Bunkyo-ku, Tokyo, 113-0033 Japan

**Keywords:** Access to medical care, Procedural justice, Interactional justice, Longitudinal studies

## Abstract

**Background:**

Using a 1-year prospective design, we examined the association of organizational justice (i.e., procedural justice and interactional justice) with refraining from seeking medical care (RSMC) among Japanese employees.

**Methods:**

We surveyed 2695 employees (1994 men and 701 women) from two factories of a manufacturing company in Japan. A self-administered questionnaire comprising scales for measuring organizational justice (Organizational Justice Questionnaire) and potential confounders (i.e., demographic and socioeconomic characteristics as well as health-related behaviors) was administered at baseline (from April to June 2011). At 1-year follow-up (from April to June 2012), a single-item question was used to measure RSMC during the follow-up period. Multiple logistic regression analysis was conducted by gender.

**Results:**

After adjusting for potential confounders, low procedural justice and low interactional justice at baseline were found to be significantly associated with higher odds of RSMC during the 1-year follow-up for male employees (odds ratio = 1.33 [95% confidence interval = 1.16–1.52], *p* < 0.001 and 1.15 [95% confidence interval = 1.02–1.29], *p* = 0.019, respectively). Similar patterns were observed for female employees (odds ratio = 1.37 [95% confidence interval = 1.08–1.74], *p* = 0.009 and 1.23 [95% confidence interval = 1.02–1.50], *p* = 0.035 for low procedural justice and low interactional justice, respectively).

**Conclusions:**

The present study provided evidence that the lack of organizational justice is positively associated with RSMC among Japanese employees, independently of demographic and socioeconomic characteristics as well as of health-related behaviors.

## Introduction

Access to medical care is a fundamental human right and an important determinant of health [1]. The effects of delayed access to medical care on reduced quality of life, longer hospital stays, and mortality have been reported across a wide range of age groups [2–5]. In Europe and Oceania, 7–22% of adults reportedly refrain from seeking medical care (i.e., are reluctant to seek medical care) for financial reasons [6]. In Japan, where people enjoy universal health insurance coverage (the co-payment rate for the working-age population is 30%) [7], about one quarter of people have been reported to refrain from seeking medical care for the same reasons [8], which is the second-highest level among high-income countries following the USA [6]. Several studies of community residents have reported that social class (i.e., educational attainment, household income, and employment conditions) [9–14] as well as regional environmental factors (i.e., community size, having some means of transportation, non-familial support, and social capital in the neighborhood) [1, 15–18] have an effect on refraining from seeking medical care (RSMC). On the other hand, work environmental factors may play an equally important role in influencing individual’s RSMC, because most of the world’s population (58%) spends one third of their adult life at work [19].

Organizational justice may be one of the important factors determining RSMC among employed people. It has its origins in human rights theory and can be defined as an employee’s perception of the fairness of resource allocation in the workplace organization [20, 21], which refers to management’s decisions and actions that are morally right and are in accordance with ethical standards and/or law [22]. In the last two decades, it has been considered as one of the psychosocial determinants of health-related behaviors and health outcomes in occupational settings [23–26]. Among others, procedural justice (i.e., the degree to which fair decision-making procedures are used to arrive at a decision [27] according to six fair process criteria, such as consistency, lack of bias, correctability, representation, accuracy, and ethicality [28]) and interactional justice (i.e., the degree to which employees are treated with respect, kindness, and dignity in interpersonal interactions with supervisor, sometimes known as interpersonal justice, and the adequacy of the explanations in terms of their timeliness, specificity, and truthfulness, sometimes known as informational justice) [29] have been viewed as primary characteristics of organizational justice within a workplace [30].

Given the definition of procedural justice and interactional justice described above [27–29], employees are less likely to be accepted as unique individuals and their fundamental human rights are less likely to be respected when organizational justice is lacking. In such a situation, employees may be mistreated just because they seek medical care and/or they may have difficulty consulting with their supervisor about re-arranging their schedules associated with seeking medical care; hence, they may refrain from seeking necessary medical care even when getting sick [20].

From the viewpoint of behavioral medicine, seeking medical care (or medical care utilization) is driven by help-seeking (or health-seeking) behavior (HSB) [31], which refers to a sequence of remedial actions that individuals undertake to rectify perceived ill-health [32]. Conceptually, the antecedents of HSB include psychosocial factors [33] as well as predisposing factors, such as workplace stress factors [34], which are postulated to influence an individual’s decision to seek initial and continued care for their perceived health issue. A recent study reported that organizational justice is positively associated with employees’ HSB [35]. Given such a conceptual framework and the empirical findings, employees who perceive lower levels of organizational justice may have difficulty making a decision to take help-seeking action because they are less likely to feel that they have a voice in or are respected by their workplace and/or supervisor, which may in turn lead to RSMC. To the best of our knowledge, the association of organizational justice with RSMC has not been examined.

For other work environmental factors, low job control has been reported to be associated with having less access to medical care among Japanese male employees, although it was specific to one situation (i.e., after diabetes screening in the workplace) [36]. This empirical finding also suggests that organizational justice has a potential effect on RSMC because it captures more basic elements of the social structure within which task-level job characteristics, such as job demands and job control, are operating [37].

The purpose of the present study was to examine the association of organizational justice (i.e., procedural justice and interactional justice) with RSMC among Japanese employees using a 1-year prospective design. It was hypothesized that those who perceived lower levels of organizational justice at baseline would be more likely to refrain from seeking medical care during the 1-year follow-up. In our analysis, we considered the existing evidence indicating that women experience more gender discrimination in the workplace than do men [38]. In fact, our previous study of Japanese employees revealed that female employees perceived lower levels of organizational justice than did male employees [39]. In Japan’s male-dominated workplace culture, female employees may have little voice in the workplace, which may lead to gender difference in the association of organizational justice with RSMC. Therefore, the analysis was conducted separately for male and female employees.

## Methods

### Study Design

In the present study, we used a part of the longitudinal data collected in an occupational cohort study on social class and health in Japan (Japanese Study of Health, Occupation, and Psychosocial Factors Related Equity: J-HOPE) at baseline (from April to June 2011) and 1-year follow-up (from April to June 2012) [40].

### Participants

All employees from two factories of a manufacturing company in Japan (*N* = 3630) were recruited by means of an invitation letter sent by the authors in February 2011. It should be noted that they were covered by the same corporate health insurance. Furthermore, because the two factories were located close to each other, the employees had almost equal access to medical care. All variables used in the present study, except employment status, which was obtained from the personnel records of the surveyed company, were measured using a self-administered questionnaire. Overall, 3461 employees completed the self-administered questionnaire at baseline (response rate 95.3%). During the 1-year follow-up period, 336 out of 3461 employees were transferred to other sites, took a leave of absence (i.e., sick leave, maternity leave, or childcare leave), retired, or declined to participate. Overall, 3125 employees participated at 1-year follow-up and completed the follow-up questionnaire (follow-up rate 90.3%). After excluding 430 employees who had at least one missing response for variables relevant to the present study, the data from 2695 employees (1994 men and 701 women) were analyzed. The analysis was conducted using the J-HOPE first and second wave datasets as of December 22, 2016.

### Measures

#### Exposure: Organizational Justice (Baseline Survey)

Organizational justice was measured using the Japanese version of the Organizational Justice Questionnaire (OJQ) [41–43], which comprises a seven-item procedural justice scale and a six-item interactional justice scale, both measured on a five-point Likert-type scale ranging from “1 = *strongly disagree*” to “5 = *strongly agree*.” The total score for each OJQ subscale was calculated by averaging item scores (score range 1–5). In this sample, Cronbach’s alpha coefficients were 0.88 for the procedural justice scale and 0.94 for the interactional justice scale for male employees; and 0.90 for the procedural justice scale and 0.95 for the interactional justice scale for female employees.

#### Outcome: RSMC (1-Year Follow-Up Survey)

In the follow-up questionnaire, we included a single-item question measuring RSMC, which was used in the Japanese General Social Survey conducted in 2008 (JGSS-2008) [13]. The participants were asked to respond to the question: “In the past year, have you ever refrained from visiting a hospital, clinic, acupuncturist, or dentist despite your sickness (including a slight cold or cavity) or injury?” The response options were “1 = *Yes, I have*,” “2 = *No, I have not*,” and “3 = *I did not get sick or injured*.” Participants were dichotomized into those who refrained from seeking medical care (i.e., those who answered 1) and those who did not (i.e., those who answered 2 or 3).

#### Potential Confounders (Baseline Survey)

Potential confounders included demographic characteristics, socioeconomic characteristics, and health-related behaviors. Demographic characteristics included age, past medical history, household size, work shift, and working hours per week. Socioeconomic characteristics included education, equivalent annual household income, occupational position, and employment status. For equivalent annual household income, the participants were asked to report their annual household income by selecting one of the following six response options: 2.99 million JPY (36,000 USD) or less, 3–4.99 million JPY (36,100–60,100 USD), 5–7.99 million JPY (60,200–96,300 USD), 8–9.99 million JPY (96,400–120,400 USD), 10–14.99 million JPY (120,500–180,600 USD), and 15 million JPY (180,700 USD) or more (USD was converted from JPY using monthly exchange rate as of April 2011 [83 JPY per USD]). Subsequently, equivalent household income was calculated by dividing the median household income of each response option by the square root of the household size. Health-related behaviors included smoking habits (never smoker, ex-smoker, and current smoker), drinking habits (rarely, sometimes, and daily), and physical activity (none, light physical activity one or more times a week, intense physical activity once or twice a week, and intense physical activity thrice or more times a week). Categories of demographic and socioeconomic characteristics are shown in Table 1.

**Table 1:** Detailed characteristics of employees who participated in the present study

	Men (*n* = 1994)					Women (*n* = 701)				
	Refrained from seeking medical care (*n* = 936)		Did not refrain from seeking medical care (*n* = 1058)		*p* value^a^	Refrained from seeking medical care (*n* = 290)		Did not refrain from seeking medical care (*n* = 411)		*p* value^a^
	Mean (SD)	*n* (%)	Mean (SD)	*n* (%)		Mean (SD)	*n* (%)	Mean (SD)	*n* (%)	
Age	37.2 (10.7)		39.2 (11.3)		< 0.001	39.2 (10.1)		42.5 (10.2)		< 0.001
29 years or less		273 (29.2)		257 (24.3)			64 (22.1)		65 (15.8)	
30–39 years		286 (30.6)		279 (26.4)			71 (24.5)		78 (19.0)	
40–49 years		240 (25.6)		313 (29.6)			111 (38.3)		152 (37.0)	
50–59 years		122 (13.0)		171 (16.2)			41 (14.1)		107 (26.0)	
60 years or more		15 (1.6)		38 (3.6)			3 (1.0)		9 (2.2)	
Past medical history^b^					0.532					0.144
Any		236 (25.2)		254 (24.0)			57 (19.7)		100 (24.3)	
None		700 (74.8)		804 (76.0)			233 (80.3)		311 (75.7)	
Work shift					0.796					0.166
Day shift		553 (59.1)		643 (60.8)			246 (84.8)		368 (89.5)	
Shift work with night duty		309 (33.0)		340 (32.1)			7 (2.4)		5 (1.2)	
Shift work without night duty		60 (6.4)		63 (6.0)			6 (2.1)		3 (0.7)	
Night shift		14 (1.5)		12 (1.1)			31 (10.7)		35 (8.5)	
Working hours per week					0.471					0.004
30 h or less		62 (6.6)		77 (7.3)			159 (54.8)		274 (66.7)	
31–40 h		188 (20.1)		245 (23.2)			82 (28.3)		90 (21.9)	
41–50 h		418 (44.7)		448 (42.3)			40 (13.8)		31 (7.5)	
51–60 h		207 (22.1)		219 (20.7)			8 (2.8)		10 (2.4)	
61 h or more		61 (6.5)		69 (6.5)			1 (0.3)		6 (1.5)	
Education					0.224					0.016
Graduate school		104 (11.1)		119 (11.2)			7 (2.4)		- (0.0)	
College		142 (15.2)		197 (18.6)			7 (2.4)		8 (1.9)	
Junior college		160 (17.1)		172 (16.3)			67 (23.1)		101 (24.6)	
High school or junior high school		530 (56.6)		570 (53.9)			209 (72.1)		302 (73.5)	
Equivalent annual household income^c^	46,180 (20,650)		47,747 (20,157)		0.085	36,892 (21,048)		36,578 (22,253)		0.848
Occupational position					0.123					0.004
Manager		107 (11.4)		140 (13.2)			- (0.0)		- (0.0)	
Non-manual employee		270 (28.8)		303 (28.6)			90 (31.0)		84 (20.4)	
Manual employee		486 (51.9)		508 (48.0)			112 (38.6)		170 (41.4)	
Others		73 (7.8)		107 (10.1)			88 (30.3)		157 (38.2)	
Employment status					0.763					0.006
Permanent employee		924 (98.7)		1046 (98.9)			132 (45.5)		145 (35.3)	
Non-permanent employee		12 (1.3)		12 (1.1)			158 (54.5)		266 (64.7)	
Procedural justice (1–5)	3.11 (0.66)		3.24 (0.68)		< 0.001	3.04 (0.72)		3.18 (0.65)		0.008
Interactional justice (1–5)	3.45 (0.80)		3.53 (0.78)		0.025	3.33 (0.85)		3.43 (0.80)		0.097

^a^Student’s *t* test and Fisher’s exact test were used for continuous and categorical variables, respectively

^b^Defined as having past medical history of stroke, myocardial infarction, hypertension, diabetes, hyperlipidemia, cancer, or mental disorders

^c^Currency unit is USD, which was converted from JPY using monthly exchange rate as of April 2011 (83 JPY per USD)

### Sample Size

Multiple logistic regression analysis was selected as a main analysis. According to a formula proposed by Peduzzi et al. [44], we calculated the minimum required sample size for multiple logistic regression analysis while considering that the prevalence of RSMC among Japanese employees has been reported to be about 50% for both genders [45] and that the maximum number of independent variables (i.e., the number of continuous variables and dummy variables in the fully adjusted model) was 29 for male and 28 for female employees. As a result, the minimum required sample size was 580 for male and 560 for female employees; therefore, our sample size was considered to have sufficient statistical power for the main analysis.

### Statistical Analysis

After descriptive analysis using Student’s *t* test or Fisher’s exact test, which aimed to compare those who did and those who did not refrain from seeking medical care in demographic and socioeconomic characteristics as well as in total score for each justice dimension, we conducted the main analysis. Prior to the main analysis, total score for each justice dimension was reverse-coded so that higher scores indicated lower justice, which allowed us to interpret the results easier. Taking reversed total score for each justice dimension as an independent variable, multiple logistic regression analysis was conducted to estimate the odds ratio (OR) and its 95% confidence interval (CI) for RSMC associated with a one-point decrease in each justice dimension. In the series of analysis, we first adjusted for demographic characteristics (Model 1). Subsequently, we incrementally adjusted for socioeconomic characteristics (Model 2) and health-related behaviors (Model 3). The level of significance was 0.05 (two-tailed). The statistical analysis was conducted using IBM^®^ SPSS^®^ Statistics Version 23.0 for Windows.

## Results

Table 1 shows the detailed characteristics of the participants by those who did and those who did not refrain from seeking medical care and by gender. Male employees who refrained from seeking medical care were significantly younger and had a lower perception of procedural justice and interactional justice compared to those who did not. Female employees who refrained from seeking medical care were significantly younger and highly educated, worked longer hours, had a greater proportion of non-manual employees and permanent employees, and had a lower perception of procedural justice compared to those who did not. Furthermore, female employees who refrained from seeking medical care had significantly larger household size compared to those who did not (mean [standard deviation] = 3.77 [1.65] and 3.51 [1.61], respectively, *p* = 0.038).

Table 2 shows the results of the multiple logistic regression analysis. For male employees, after adjusting for demographic characteristics (Model 1), low procedural justice was significantly associated with higher odds of RSMC (*p* < 0.001) in that a one-point decrease in procedural justice led to a 1.34 (95% CI 1.17–1.53)-fold increase in the odds of RSMC. Similarly, low interactional justice was significantly associated with higher odds of RSMC (*p* = 0.013) in that a one-point decrease in interactional justice led to a 1.16 (95% CI 1.03–1.30)-fold increase in the odds of RSMC. These patterns remained unchanged after additionally adjusting for socioeconomic characteristics and health-related behaviors (Models 2 and 3).

**Table 2:** Association of low organizational justice with refraining from seeking medical care at 1-year follow-up among Japanese employees by gender: the results of multiple logistic regression analysis

	Model 1^a^		Model 2^b^		Model 3^c^	
	OR (95% CI)	*p* value	OR (95% CI)	*p* value	OR (95% CI)	*p* value
Men (*n* = 1994)						
Procedural justice	1.34 (1.17–1.53)	< 0.001	1.35 (1.18–1.55)	< 0.001	1.33 (1.16–1.52)	< 0.001
Interactional justice	1.16 (1.03–1.30)	0.013	1.15 (1.02–1.29)	0.018	1.15 (1.02–1.29)	0.019
Women (*n* = 701)						
Procedural justice	1.39 (1.11–1.76)	0.005	1.36 (1.08–1.72)	0.010	1.37 (1.08–1.74)	0.009
Interactional justice	1.21 (1.00–1.46)	0.054	1.23 (1.02–1.50)	0.035	1.23 (1.02–1.50)	0.035

In the analysis, total scores for procedural justice and interactional justice were reverse-coded so that higher scores indicated lower justice

*OR* odds ratio, *CI* confidence interval

^a^Adjusted for age, past medical history, household size, work shift, and working hours per week

^b^Additionally adjusted for education, equivalent annual household income, occupational position, and employment status

^c^Additionally adjusted for smoking habits, drinking habits, and physical activity

For female employees, after adjusting for demographic characteristics (Model 1), low procedural justice was significantly associated with higher odds of RSMC (*p* = 0.005) in that a one-point decrease in procedural justice led to a 1.39 (95% CI 1.11–1.76)-fold increase in the odds of RSMC. This pattern remained unchanged after additionally adjusting for socioeconomic characteristics and health-related behaviors (Models 2 and 3). On the other hand, although low interactional justice was associated with higher odds of RSMC, the result was not statistically significant (*p* = 0.054) after adjusting for demographic characteristics (Model 1). However, after additionally adjusting for socioeconomic characteristics and health-related behaviors (Models 2 and 3), this association became significant (*p* = 0.035) in that a one-point decrease in interactional justice led to a 1.23 (95% CI 1.02–1.50)-fold increase in the odds of RSMC.

## Discussion

The present study demonstrated a significant association of low procedural justice and low interactional justice at baseline with RSMC during the 1-year follow-up for male employees, even after adjusting for demographic and socioeconomic characteristics as well as for health-related behaviors. For female employees, similar patterns were observed, with an exception of non-significant association of low interactional justice with RSMC after adjusting for demographic characteristics.

Our results showed that low procedural justice was significantly associated with RSMC for both genders, which supported our hypothesis. In Japan, it is common to take time off (i.e., paid holiday) to seek medical care on working days because paid sick leave is not stipulated by law. In principle, it is possible for employees to take time off without explaining their reasons, while workplaces also have a right to ask employees about the reasons for taking time off to maintain normal business operations. Regardless of reasons, workplaces should not treat employees who want to take time off unfairly. However, in work settings in which decision-making styles are unfair and obscure, employees may be afraid of being mistreated just because they take time off [46], which may make them to have difficulty seeking necessary medical care. Furthermore, a significant association of low interactional justice with RSMC was observed in the fully adjusted model for both genders, which also supported our hypothesis. When employees perceive the attitude of their supervisor as irreverent, they may face difficulties consulting with him/her about taking time off to seek medical care and re-arranging their work schedules. From the viewpoint of behavioral medicine, HSB may be a key mediator of the association of organizational justice with RSMC. As introduced earlier, organizational justice has been reported to be positively associated with employees’ HSB [35]. In work settings in which organizational justice is lacking, employees are less likely to perceive that they have a voice in or are respected by their workplace and/or supervisor. Such perception of injustice may repress their decision making to take help-seeking action, which may lead to RSMC. Future research on detailed mechanisms underlying the association of organizational justice with RSMC is needed.

When we compare the strength of the association of procedural justice with RSMC with that of interactional justice, procedural justice had a greater association with RSMC. This could be attributed to the fact that procedural justice is more closely related to company regulations that stipulate employees’ time off and sickness absence. Our findings suggest that procedural justice rather than interactional justice is a stronger determinant of medical care seeking behavior among employees.

Although the strength of the association of procedural justice with RSMC was similar for male and female employees, the association of interactional justice with RSMC was slightly greater for female employees than for male employees. This gender difference may be explained by the fact that all managers were men in our sample (see Table 1); hence, our female participants always had to interact with a supervisor of the opposite gender. Pelled and Xin [47] reported that employees show higher levels of trust and relationship quality in same-gender supervisory relationships than in opposite-gender ones. Therefore, in our sample, female employees may be more hesitant to discuss taking time off to seek medical care with their male supervisor, especially with regard to female-specific diseases, when they perceive him as having low interactional justice. The imbalanced male-female ratio of managers observed in our sample is common in the male-dominated workplace culture in Japan. In fact, the latest national statistics on employment in Japan have reported that the average proportion of female managers is still only about 10%, and about 45% of companies do not have any female manager [48]; therefore, our findings may be true of many other Japanese companies. However, the association of interactional justice with RSMC in the context of other types of supervisory relationships, such as female supervisor–male employee or female supervisor–female employee relationships, should be examined in future research.

Furthermore, for female employees, the association of interactional justice with RSMC was not significant after adjusting for demographic characteristics (Model 1), while it became significant after additionally adjusting for socioeconomic characteristics (Model 2). According to Table 1, female employees who refrained from seeking medical care had relatively higher socioeconomic status. Highly educated and/or permanent employees are more likely to be expected to play an important role in their workplace and therefore to be respected by supervisor. At the same time, such pressure from the workplace may make it difficult for them to seek medical care when they get sick. Such a background may be reflected in our findings of the association of interactional justice with RSMC for female employees.

Possible limitations of the present study should be considered. First, some employees dropped out at follow-up due to sick leave. These employees may have perceived lower levels of organizational justice at baseline and refrained from seeking medical care until their disease became severe, which may have underestimated the true association. Furthermore, 430 out of 3125 employees were excluded from the analysis due to missing responses. It has been reported that the lack of organizational justice is associated with poor mental health, such as psychiatric disorders and depression [24], which present with poor concentration. Therefore, those who perceived lower levels of organizational justice may have been more likely to have missing responses due to poor concentration and to be excluded from the analysis. Such excluded employees may have been highly encouraged to seek medical care due to severe psychological symptoms. Our results may thus have overestimated the true association. Second, we measured RSMC by simply asking the participants to recall their experience over the past year; therefore, recall bias may have skewed our findings. Furthermore, we focused only on refraining from seeking “therapeutic” care when individuals get sick but not on “preventive” care, such as regular dental care. Further research on RSMC should also focus on preventive care. Third, RSMC at baseline may have affected our findings, as it may have been influenced by personality traits. Recent studies have reported that neuroticism is associated with an increased number of physician visits [49] as well as with lower levels of perceived justice [50]; therefore, our findings may be underestimated. Fourth, although we conducted the gender-stratified analysis, the distribution of socioeconomic characteristics was quite different between genders. Especially for employment status, almost all men were permanent employees, while the proportion of permanent employees among women was only 40% (see Table 1). It is possible that organizational justice is maintained only among permanent employees [51]. Therefore, such a difference in the distribution of employment status across genders might have affected our findings. Fifth, our data was obtained from one particular manufacturing company in Japan from 2011 to 2012; therefore, there is a limitation to generalizability and some changes in context may have occurred for the last 6 to 7 years. Our findings should thus be interpreted with caution. Sixth, organizational justice is defined as an employee’s “perception” of the fairness in the workplace. However, perceived stress measured by self-report has been reported to be only moderately related to actual stress exposure [52]. Therefore, our findings do not completely reflect the association of actual exposure to organizational (in)justice with RSMC. Finally, although a recent study on organizational justice utilized a multilevel approach in view of its contextual effect [53], the present study could not examine such an effect.

In conclusion, the present study provided evidence that the lack of procedural justice increases the tendency to refrain from seeking medical care among Japanese employees, independently of demographic and socioeconomic characteristics as well as of health-related behaviors. Our findings suggest that establishing fair and open decision-making styles in the workplace effectively promotes medical care-seeking behaviors among employees. Although interactional justice, characterized by the fair and respectful attitude of the supervisor, may also be an important factor associated with RSMC, future studies on this topic should account for gender differences in supervisory relationships.
